# Patterns of Cognitive Dysfunction in Progressive MS

**DOI:** 10.3233/BEN-120286

**Published:** 2013

**Authors:** Peter Connick, Siddharthan Chandran, Thomas H. Bak

**Affiliations:** ^1^Department of Clinical Neurosciences, University of Cambridge, CambridgeUK; ^2^Centre for Clinical Brain Science, University of Edinburgh, EdinburghUK; ^3^School of Philosophy, Psychology and Language Sciences, University of Edinburgh, EdinburghUK

**Keywords:** Multiple sclerosis, clinical trials observational study, assessment of cognitive disorders/dementia, neuropsychological assessment

## Abstract

**Background:** Progressive MS is associated with a high frequency of cognitive impairment. However, it is not clear to what extent this reflects global dysfunction, or independent deficits in specific functions.

**Objective:** To characterise patterns of cognitive impairment in progressive MS on a multi-dimensional cognitive assessment tool well established in neurodegenerative diseases.

**Methods:** Patients with secondary (SPMS; *n* = 60) and primary progressive MS (PPMS; *n* = 28) were assessed using the Addenbrooke’s Cognitive Examination-Revised (ACE-R) multi-dimensional cognitive assessment scale. Independent dimensions of impairment and their relative contribution to the overall burden of cognitive dysfunction were then determined by factor analysis.

**Results:** Two independent dimensions of impairment were seen: frontal-executive (attention, verbal fluency, recall) on one hand, and language and visuospatial functions on the other. These accounted for 55% and 45% respectively of the variance not explained by a global influence (14.2% and 11.6% respectively of total variance). Isolated language and visuospatial dysfunction was seen in both groups, whereas isolated impairment in frontal-executive functions was underrepresented in SPMS (*p* = 0.001) and not seen in PPMS patients (*p* = 0.040).

**Conclusions:** In addition to a prominent global influence on cognitive performance, patients with progressive MS commonly exhibit language and visuospatial deficits. Evaluation of these abilities should therefore be included in clinical assessment of cognition in progressive MS.

## 1. Introduction

Multiple sclerosis (MS) is the commonest non-traumatic cause of acquired disability amongst young European adults [1], with cognitive impairment seen in 40–70% of cases [2]. Reduced speed of information processing, together with impairment in executive functions, recall memory, and attention are the most frequently described deficits [3,4], particularly in the most extensively studied patient group with relapsing-remitting disease. In comparison, language and visuospatial functions have received less attention, despite evidence of involvement in large cohort studies [5] as well as in studies specifically assessing these domains [6,7]. It therefore remains unclear whether cognitive dysfunction in MS always reflects a universal deficit of all functions, or whether impairment of specific functions can occur independently. The question has practical implications for the choice of appropriate assessment instruments. In the first case, the cognitive impairment is best measured by current batteries, focused on the speed of information processing and ex-

ISSN 0953-4180/13/$27.50 © 2013 – IOS Press and the authors. All rights reserved ecutive functions [8,9]. In the second case, preference should be given to tests distinguishing specific impairments of individual cognitive domains.

This issue is particularly relevant for the less-researched progressive forms of the disease. A neurodegenerative component of pathology is increasingly recognized in all forms of MS, but may be particularly salient to the progressive phase [10,11]. Neuroaxonal damage and loss in the context of progressive disease therefore represents a possible pathological basis for differences in the clinical features of cognitive impairment between patients with (early) relapsing-remitting disease and those in the progressive phase. Indeed, previous studies in this area suggest that progressive disease may be particularly associated with impairments in visuospatial abilities and information processing speed [12–14].

Growing recognition of a neurodegenerative component in MS pathology [15], also raises the question whether research on cognitive aspects of MS could benefit from the insights gained in recent years through the study of other neurodegenerative diseases, particularly in terms of defining specific patters of cognitive impairment characterising individual disease entities [16–18]. However, one of the main difficulties in comparing MS with neurodegenerative syndromes is the different assessment tools used in these conditions. We therefore decided to examine patients with progressive MS using one of the best established and most successful cognitive screening tools used in neurodegeneration: the Addenbrooke's Cognitive Examination Revised (ACE-R). The ACE-R is a sensitive clinical tool for the multi-domain assessment of cognition, including language and visuospatial functions [19]. It comprises a 100-point assessment of orientation and attention (18-points), memory (26-points), verbal fluency (14-points), language (26-points), and visuospatial function (16-points). The ACE-R is a practical bedside tool that has been validated and widely applied for the assessment of cognition in neurodegenerative diseases with cortical and subcortical pathology including Alzheimer's disease [16], frontotemporal dementia [16], dementia with Lewy bodies [17], primary progressive aphasia [20], multisystem atrophy, corticobasal degeneration, and progressive supranuclear palsy [18]. Studies comparing performance with another well-established cognitive screening tool, the Dementia Rating Scale (DRS), demonstrated that ACE, despite being shorter and easier to use, reached equal levels of sensitivity in detecting cognitive impairment [17,18]. By using multivariate statistical analysis, we evaluated the contribution of global dysfunction and dysfunction in specific mental abilities to the overall pattern of cognitive impairment in progressive MS.

## 2. Methods

Participants were recruited from a research clinic for patients with progressive MS in Cambridge, UK. Approval was obtained from the local ethics committee and all patients gave written informed consent.

### 2.1. Recruitment of subjects

Patients were recruited from the East Anglia and North London regions of the UK. Eligibility criteria were: revised (2005) McDonald criteria MS [21] and a progressive disease course. Eighty-seven control subjects without known neurological or psychiatric disorder were also recruited locally. A matched control cohort was then achieved through stratified sampling by age, gender and educational level ($n = 24$).

### 2.2. Assessment schedule

Patients were assessed at a single visit by neurological history and examination. Data was not collected on previous disease modifying therapy use. Cognitive function was evaluated using the Addenbrooke's Cognitive Examination (Revised) (ACE-R). All cognitive assessments were performed by PC. In brief, assessment of each cognitive domain comprised the following:

*Attention and orientation*: (18 points) Orientation in time and space, registration of three words and serial subtraction/backwards spelling.

*Memory*: (26 points) Recall of three words (1 minute) and recall of fictitious name and address (immediate and after 10 mins), and four general knowledge questions.

*Verbal fluency*: (14 points) Spontaneous generation of words over one minute defined by initial letter (P) and semantic category (animals).

*Language*: (26 points) Written and verbal comprehension (one and three-step), writing ability, repetition of words and phrases, object-recognition and naming, reading ability.

*Visuospatial*: (16 points) Copying of overlapping pentagons and wire cube, generation of a clock face, dot counting and naming incomplete letters.

Table 1
Participant characteristics

| | MS disease course | | | |
|---|---|---|---|---|
| | SPMS | PPMS | All | p |
| n | 60 | 28 | 88 | – |
| Age (mean, SD) | 50.8 (8.8) | 51.3 (11.2) | 50.9 (9.6) | 0.54 |
| Male sex (%) | 56.7 | 53.6 | 55.7 | 0.79* |
| Years in full-time education (mean, SD) | 13.1 (2.9) | 12.6 (2.7) | 12.9 (2.8) | 0.43 |
| Disease duration in years (mean, SD) | 13.6 (7.8) | 9.4 (5.6) | 12.3 (7.4) | 0.02 |
| Total MSIS-29 (mean, SD) | 85.3 (21.6) | 84.4 (21.7) | 85 (21.5) | 0.57 |
| MSIS-29 physical sub-score (mean, SD) | 63.0 (16.8) | 62.9 (15.7) | 63.0 (16.3) | 0.69 |
| MSIS-29 psychological sub-score (mean, SD) | 22.3 (7.6) | 21.5 (8.2) | 22.0 (7.8) | 0.41 |
| EDSS (mean, SD) | 5.9 (1.2) | 5.7 (1.5) | 5.8 (1.3) | 0.43 |
| BDI-II (mean, SD) | 15.3 (14.0) | 14.2 (9.6) | 14.9 (12.7) | 0.89 |
| PASAT score (mean, SD) | 39.0 (12.1) | 35.0 (14.1) | 37.8 (12.7) | 0.18 |
| ACE-R score (mean, SD) | 91.6 (7.8) | 89.4 (8.3) | 90.9 (8.3) | 0.08 |

SD = Standard deviation, MSIS-29 = Multiple Sclerosis Impact Scale 29, EDSS = Expanded Kurtzke Disability Status Scale, BDI-II = Beck's Depression Index (II), ACE-R = Addenbrooke's Cognitive Examination (Revised), PASAT = Paced Auditory Serial Addition Test. Significance tests shown for equivalence between SPMS and PPMS, performed by Mann-Whitney except (*) = Pearson's $\chi^2$ test.

Further assessment included: The multiple sclerosis impact scale-29 (MSIS-29), Beck depression inventory (II) (BDI-II), expanded disability status scale (EDSS), and the 3-second paced auditory serial additions test (PASAT).

### 2.3. Statistics

Analyses were performed using Stata SE (Version 9, Stata Corp. TX, USA). Group comparisons for participant characteristics were performed using the Mann-Whitney U-test and Pearson's $\chi^2$ test. Independent dimensions of cognitive impairment were determined by exploratory factor analysis using ACE-R domain scores as items., Pearson's product moment correlations between domains were calculated and the correlation matrix inspected for ill-conditioning (defined as intercorrelation $> 0.8$) prior to further analysis. The correlation matrix was also tested for singularity by Bartlett's test of sphericity and sampling adequacy confirmed by the Kaiser-Meyer-Olkin test (adequacy defined by KMO $> 0.6$ for individual domains and whole model). Factor extraction was performed using the principal factor method with factors retained following inspection of the screeplot [22]. In order to improve interpretability while allowing for correlation between retained factors, an oblique rotation was used (direct quartimin). Factor scores were generated by the regression method [23], and internal consistency was assessed by Cronbach's alpha. Higher order structure was also evaluated by the principle factor method. Assessment of latent general factor contributions was performed by Schmid-Leiman orthogonal transformation using computational methods described by Thomson in the MacOrtho software package [24,25]. The proportion of test variance attributable to higher and first-order factors was assessed by McDonald's $\omega_h$ measure [26]. Data are shown as mean ± SD unless otherwise stated.

## 3. Results

### 3.1. Participant characteristics

Case and control groups were matched for age (50.9 ± 9.6 *vs.* 52.0 ± 9.4; $p = 0.63$), sex (male = 56% *vs.* 50%; $p = 0.62$), and years in full-time education (12.9 ± 0.3 *vs.* 13.4 ± 0.7; $p = 0.51$). Sixty-eight percent (60/88) of the patient group had secondary progressive MS (SPMS) and thirty-two percent (28/88) primary progressive MS (PPMS). No patient had experienced a relapse or received treatment with steroids in the 3-months prior to assessment, and no patients were excluded from the study. The SPMS group had a higher mean disease duration than the PPMS group (13.6 ± 7.8 years *vs.* 9.4 ± 5.6 years; $p = 0.02$), but there were no other differences in demographic or disease characteristics; or in cognitive performance assessed by the ACE-R and PASAT (Table 1).

### 3.2. Independent dimensions of cognitive impairment

In order to determine if independent dimensions of cognitive dysfunction were present, exploratory factor analysis was performed using ACE-R domain scores as items. This revealed two dimensions of covariance that showed significant emphasis for frontal-executive

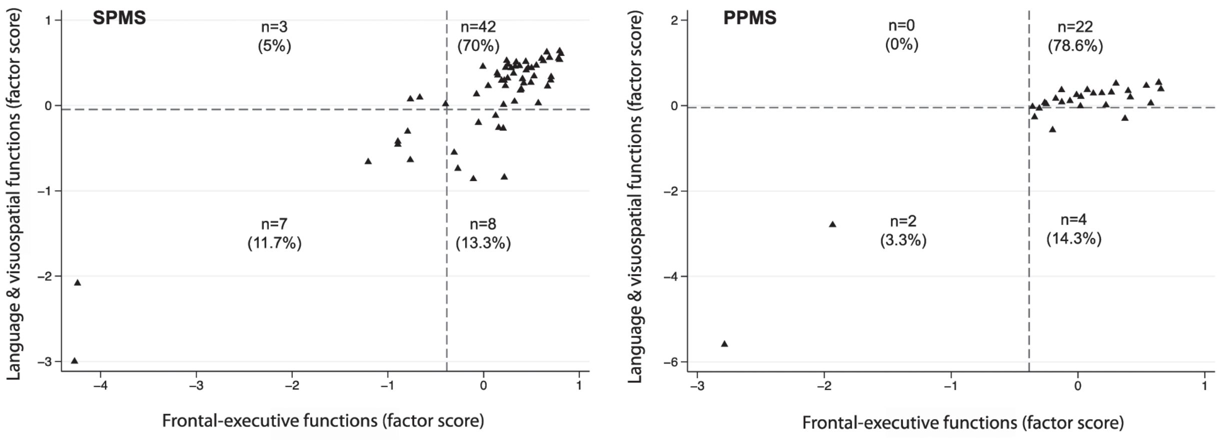

Fig. 1. Patterns of cognitive impairment by disease course. Scatterplots for scores of individual patients on the two dimensions of cognitive impairment identified by factor analysis (D1 & D2). Dashed lines indicate reference ranges for healthy controls (2 SD below the control mean). Patients in both disease groups show an excess of impairment for language and visuospatial functions (D2), with isolated frontal-executive impairment (D1) rarely seen (SPMS, Fisher's exact, $p = 0.001$; PPMS, Fisher's exact, $p = 0.040$).

Table 2
First-order factor analysis of cognitive domain scores

| | D1 | D2 |
|---|---|---|
| Attention and orientation | **0.72** | 0.10 |
| Fluency | **0.62** | −0.10 |
| Memory | **0.75** | −0.01 |
| Language | 0.01 | **0.75** |
| Visuospatial | 0.00 | **0.80** |
| Eigenvalue | 2.25 | 0.41 |

Two independent dimensions of cognitive impairment are shown with high factor loading for attention and orientation, fluency, and recall memory (D1); and language and visuospatial functions (D2) respectively. ACE-R = Addenbrooke's Cognitive Examination (Revised).

functions ("D1": *Attention and Orientation*, *Memory,* and *Verbal fluency*), and separately for posterior cortical functions ("D2": *Language* and *Visuospatial*; Table 2). Both dimensions had high internal consistency (Cronbach's alpha = 0.69 and 0.89 respectively). Performance on the PASAT was moderately correlated with D1 ($r = 0.53$) but weakly correlated with D2 ($r = 0.39$).

### 3.3. *Pattern of cognitive impairment*

Scatterplots of subject-scores for the two independent dimensions of impairment were examined to determine if impairment occurred in a predictable pattern with respect to primary and secondary progressive patient groups. A consistent distribution of impairment was seen (figure), with isolated impairment in posterior cortical functions overrepresented in both SPMS and PPMS groups (Fisher's exact test; $p = 0.001$ and $p = 0.040$ respectively).

Table 3
Contribution of general and independent factors to variance in cognitive functions

| | General factor | Independent factors | |
|---|---|---|---|
| | G | D1 | D2 |
| Attention and orientation | **0.698** | **0.364** | 0.048 |
| Fluency | **0.443** | **0.314** | −0.053 |
| Memory | **0.638** | **0.383** | −0.005 |
| Language | **0.659** | 0.006 | **0.383** |
| Visuospatial | **0.673** | 0.001 | **0.397** |
| % Variance explained | **74.2** | **14.2** | **11.6** |

Schmid-Leiman solution showing the relative contributions of a general factor (G) and independent factors (D1 and D2) to the variance in ACE-R cognitive domain scores observed in patients with progressive MS.

### 3.4. *The contribution of a global influence to cognitive impairment*

The contribution of a global influence in cognitive performance was then assessed by higher-order factor analysis. This showed evidence of a general factor that accounted for 74.2% of total variance in cognitive performance. Independent effects of first-order factors D1 and D2 therefore accounted for 14.2% and 11.6% of total variance respectively (Table 3).

### 3.5. *Differences in the pattern of cognitive dysfunction by disease course*

In order to compare disease groups, separate analyses for SPMS and PPMS were also performed. Screeplot inspection following initial factor extraction supported retention of one factor in both analyses. The cognitive domains with highest factor loading differed

Table 4
Shared variance in cognitive functions by disease course

| | SPMS | PPMS |
|---|---|---|
| Attention and orientation | **0.83** | **0.77** |
| Fluency | **0.67** | 0.14 |
| Memory | **0.76** | **0.62** |
| *Language* | 0.52 | **0.94** |
| Visuospatial | 0.55 | **0.92** |
| Eigenvalue | **2.28** | **2.73** |

Factor analysis of ACE-R domain sub-scores by disease course. Initial extraction supported retention of one factor in both models, unrotated factor loadings are therefore shown.

between groups, with the SPMS group having highest shared variance in attention, memory and fluency scores. In contrast, the PPMS group had highest shared variance in attention, language and visuospatial domain scores (Table 4).

## 4. Discussion

Like those in relapsing-remitting disease, studies of cognition in progressive MS have typically focused on impairment in executive functions, recall memory, and attention; a pattern viewed as archetypal of the disease [2]. Our study demonstrates the existence of two independent dimensions of impairment that account for 55% and 45% respectively of the variance in cognitive function not explained by a global influence. The first dimension (D1) reflects a well established "core impairment" of cognitive functions: attention, verbal fluency, and recall memory. Accordingly, D1 shows highest correlation with traditional measures of cognition in MS such as the Paced Auditory Serial Additions Test (PASAT). In contrast, the second dimension (D2) reflects impairment of cognitive functions rarely studied in MS: language and visuospatial abilities. Interestingly, while an isolated pattern of language and visuospatial (D2) dysfunction was observed in some patients, isolated impairment of frontal-executive (D1) functions was seen only rarely in SPMS and never in PPMS.

Our finding of a prominent global influence on cognitive performance is unsurprising when viewed in the context of a large body of research into human cognition in health. In normal individuals, a general factor ('g') accounts for around 50% of variance in performance across all psychometric tests [27]. The observed 'g' of 74.2% in our study may therefore reflect a combination of normal biology and global impairment due to disease. Information processing speed has been proposed as a possible neurobiological mechanism underpinning 'g' in healthy individuals, and a reduction of information processing speed represents an attractive interpretation of the general factor in our study given recent descriptions in healthy individuals linking 'g' to diffuse white matter tract integrity [28], a metric known to be markedly impaired in progressive MS [29]. Nevertheless, the remaining 25.8% of independent variance implies that a clinically significant burden of dysfunction falls selectively on specific cognitive functions. This independent variance was split equally between D1 and D2, indicating that their components merit equal attention in the clinical assessment of patients with suspected cognitive impairment due to MS.

As an observational study of cognitive dysfunction in progressive MS, our study has limitations. Potential confounding by physical disabilities is an issue for the evaluation of all aspects of cognition in MS – regardless of the psychometric instrument used, or the cognitive domain in question. Weakness, ataxia, spasticity, and other physical impairments may limit performance on tasks involving copying, drawing, or pointing. Reduced information processing speed may affect performance on timed tasks, dysarthria on tasks requiring verbal responses, and fatigue has the potential to impact on any complex task. These limitations apply across all existing published work in MS cognition, but are particularly pertinent to studies of patients with progressive disease. In this context, the ACE-R offers an attractive alternative to the traditional MS cognitive batteries, as it has been extensively applied to patients with neurodegenerative disorders such as PD, PSP, and MSA, who exhibit similar levels of physical disability [17,18]. Moreover, recent research suggests high sensitivity of ACE-R components to cognitive dysfunction in MS [30]. Second, despite the best efforts of regulatory authorities, underrepresentation of women in clinical research remains a significant challenge [31]. With reference to existing epidemiological UK-based data [32], women were underrepresented in our SPMS cohort (probability of the observed sex ratio = 0.02, two-sided binomial probability test). Given that all patients referred were recruited to the study, it is possible that the underrepresentation of female patients in the SPMS cohort represents bias on behalf of the referring practitioners.

Although our study does not have the necessary imaging assessments to define the precise anatomical substrate of the two independent dimensions identified by factor analysis, D1 components can be interpreted as representative of frontal-executive (FE)

functions [33–35]. More tentatively, D2 components could reflect more posterior, parieto-temporal-occipital (PTO) functions [36–39]; although it has to be remembered that frontal dysfunction can also be associated with language impairment. In our PPMS group, impairment of D2 functions was seen with or without impairment of D1, whereas D1 impairment was only seen together with impairment of D2 functions. The same distribution was also observed in the SPMS group, although rare isolated impairment in D1 functions was occasionally seen. This pattern raises the possibility that language and visuospatial impairment may be typical of cognitive dysfunction in progressive MS. Moreover, the absence of isolated frontal-executive dysfunction in our PPMS cohort is consistent with this pattern of impairment when deficits due to randomly distributed focal white matter lesions are minimal.

Our findings are of interest in the context of debate about the contribution of inflammatory processes and neurodegeneration to the clinical deficits seen in progressive MS. Although inflammation and neurodegeneration co-exist throughout the disease, they assume shifting prominence dependent upon the clinical stage, with neurodegeneration assuming greater pathogenic significance in patients with progressive disease [40]. The mechanistic relationship between these components of the disease biology remains debated [10]; however, the relative lack of focal inflammatory white matter disease in primary progressive (PPMS) compared to secondary progressive MS (SPMS) provides an opportunity to evaluate the contribution of multifocal inflammation. In addition to the absence of isolated frontal-executive impairment in our PPMS group, group-specific analysis showed low factor loading of frontal-executive functions (verbal fluency) and high loading of visuospatial and language domains; a pattern that was reversed in SPMS group. Taken together, these observations are consistent with a hypothesis that language and visuospatial impairment may reflect a predominantly neurodegenerative (or diffuse inflammatory) effect and frontal-executive dysfunction a focal inflammatory effect. Such an interpretation is supported by Huijbregts et al., who also found verbal fluency to be relatively impaired in SPMS, and spared in PPMS [13].

Our finding that language and visuospatial impairment has a significant independent contribution to overall cognitive dysfunction has important implications for the clinical assessment of patients with progressive MS. It supports a view that the traditional approach to cognitive assessment in MS, focussed on frontal-executive functions, should be complemented by assessment of more posterior cortical functions including language and visuospatial abilities. This can be achieved using comprehensive batteries such as the Minimal Assessment of Cognitive Function in MS (MACFIMS; 90 minutes to administer) [41]. However, our study also demonstrates that both frontal-executive and posterior cortical dysfunction can be identified even by a brief (20 minutes) multidimensional bedside screening tool such as the ACE-R.

## Acknowledgments

This work was supported by the Medical Research Council, The Multiple Sclerosis Society of Great Britain and Northern Ireland, and The Raymond and Beverly Sackler Foundation.

## Funding

This work was funded by the Medical Research Council and the MS Society.

## Conflict of interests

The Authors declare that there is no conflict of interest.
